# Effects of Sangu Decoction on Osteoclast Activity in a Rat Model of Breast Cancer Bone Metastasis

**DOI:** 10.1155/2012/381904

**Published:** 2012-11-06

**Authors:** Bo Deng, Li-qun Jia, Huang-ying Tan, Xuan Yao, Fu-yun Gao, Lin Pan, Jian Cui, Qing Xiang

**Affiliations:** ^1^Department of Oncology of Integrative Chinese and Western Medicine, China-Japan Friendship Hospital, Beijing 100029, China; ^2^School of Clinical Chinese Medicine, Beijing University of Chinese Medicine, Beijing 100029, China; ^3^TCM Department, Beijing Tongren Hospital, Capital Medical University, Beijing 100730, China

## Abstract

Bone metastasis (BM) is a major clinical problem for which current treatments lack full efficacy. The Traditional Chinese Medicine (TCM) Sangu Decoction (SGD) has been widely used to treat BM in China. However, no in vivo experiments to date have investigated the effects of TCM on osteoclast activity in BM. In this study, the protective effect and probable mechanism of SGD were evaluated. The model was established using the breast cancer MRMT-1 cells injected into the tibia of rat. SGD was administrated, compared with Zoledronic acid as a positive control. The development of the bone tumor and osteoclast activity was monitored by radiological analysis. TRAP stain was used to identify osteoclasts quantity and activity. TRAP-5b in serum or bone tumor and TRAP mRNA were also quantified. Radiological examination showed that SGD inhibited tumor proliferation and preserved the cortical and trabecular bone structure. In addition, a dramatic reduction of TRAP positive osteoclasts was observed and TRAP-5b levels in serum and bone tumor decreased significantly. It also reduced the mRNA expression of TRAP. The results indicated that SGD exerted potent antiosteoclast property that could be directly related to its TRAP inhibited activity. In addition it prevented bone tumor proliferation in BM model.

## 1. Background or Introduction

Bone is one of the organs liable to get remote metastasis of malignancy, with the incidence only secondary to lung and liver [1]. Bone metastases (BMs) can cause a wide range of symptoms and signs that influence the quality of life or even shorten survival [2]. Severe pain, pathological fracture, spinal cord compression, and malignant hypercalcemia represent the most important complications of skeletal involvement in patients with metastatic prostate, breast, and lung cancer [3]. At least 80% of patients who develop metastatic breast cancer will, at some point during their disease, develop BM [4]. Therefore, to control the occurrence and development of BM is one of the most important tasks for current oncologists.

**Figure 1:**
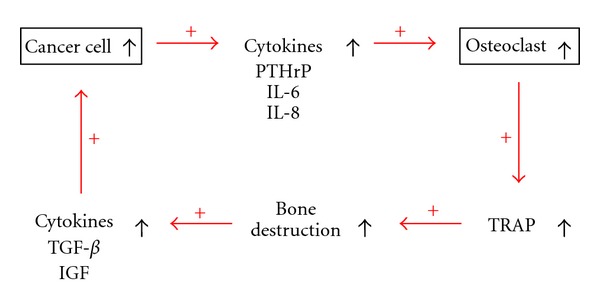
The vicious cycle of breast cancer bone metastasis.

It is thought that breast cancer cells secrete factors that act in a paracrine fashion to activate osteoclasts [5, 6]. As shown in Figure 1, breast carcinomas grow avidly in bone because the bone microenvironment provides a favorable soil. Bone destruction is mediated by osteoclasts that are stimulated by local production of the tumor peptide parathyroid hormone-related peptide (PTH-rP). Production of PTH-rP by breast carcinoma cells in bone is enhanced by growth factors produced as a consequence of normal bone remodeling, particularly activated transforming growth factor-beta (TGF-beta). Thus, a vicious cycle exists in bone between production by the tumor cells of mediators such as PTH-rP and subsequent production by bone of growth factors such as TGF-beta, which enhance PTH-rP production [7]. Tartrate-resistant acid phosphatase (TRAP) is a marker enzyme of osteoclasts [8]. TRAP-5b, a major isoform of TRAP, exists in serum and bone tumor. It is produced exclusively by osteoclasts and has been found to be correlative with osteoclast numbers. So levels of catalytically active TRAP-5b reflect bone-resorptive activity [9].

Pharmacological intervention (including bisphosphonates, radiopharmaceuticals, and opioids) is used in conjunction with other therapies (including radiotherapy, chemotherapy, hormonal therapy, and surgical intervention) to alleviate BM [10]. Traditional Chinese Medicine (TCM) includes many herbal formulae with bone preserving potential. Sangu Decoction (SGD) has been successfully used to treat cancer-induced bone pain in the clinical practice of TCM. A randomized, double-blind, placebo-controlled trial designed for observing the therapeutic effects has confirmed that SGD is effective in ameliorating cancer-induced bone pain and metastatic bone destruction [11]. Previous pharmacological studies in our laboratory demonstrated that intragastric administration of SGD significantly attenuated mechanical allodynia and thermal hyperalgesia of BM rats. The herbal preparation was also effective in improving radiological changes and preventing the bone mineral density decrease induced by BM [12].

However, no in vivo experiments to date have investigated the effects of TCM on osteoclast activity in BM and molecular targets of TCM relevant to its bone preserving effects. In this study, the molecular mechanism underlying the bone preserving like action of SGD was investigated by measuring TRAP-5b protein levels and TRAP mRNA expression in bone tumor tissues of BM rats. The objective of the present paper was to explore the potential for developing a new osteoclasts inhibitor in BM.

## 2. Materials and Methods 

### 2.1. Preparation of Sangu Decoction (SGD)

The constituents of SGD are shown in Table 1. All TCM herbs were purchased from the Pharmacy of China-Japan Friendship Hospital and were identified and authenticated by the head of the department. The voucher specimens are available in our aboratory. According to Pharmacopoeia of China (2010), extract amounts of component herbs were weighed according to the classic percentage and mixed well. The mixture was soaked in distilled water for 30 min and then boiled in 8 volumes of water (v/w) for 1 h and extracted twice. This preparation method followed the ancient method [12] and was also the same to clinical preparation. The supernatant was condensed to concentration of 3.9 g/mL by water bath. The concentration of SGD was expressed in total dry weight of the crude herbs per milliliter in decoction. 

### 2.2. Preparation of Cells

Syngeneic MRMT-1 rat mammary gland carcinoma cells were purchased from the Institute of Development, Aging and Cancer, Tohoku University (Tohoku, Japan) and prepared as described previously [13]. Cells were cultured in medium containing RPMI 1640 (Gibco, USA), 10% (50 mL) fetal bovine serum (FBS, heat-inactivated), 1% (5 mL) l-glutamine, and 2% (10 mL) penicillin/streptomycin. Cells were released from the plastic by brief exposure to 0.1% w/v trypsin. The resulting pellet was washed twice with 10 mL of Hank's balanced salt solution (HBSS, Gibco, USA) without Ca^2+^, Mg^2+^ and was finally suspended in 1 mL Hank's solution. Cells were diluted in Hank's medium to the required concentration for injection (10^6^ cells/mL) and kept on ice until injected into rats. 

### 2.3. Care of Animals

All animal procedures were conformed to Guidelines of Beijing (China) for the Ethical Use of Animals (2010). Eighty specific pathogen-free female Sprague-Dawley rats, weighing 160 ± 10 g, were provided by Beijing Weitong-Lihua Experimental Animal Technique Ltd. Co. (Certification no. SCXK (Jing) 2007-0001). Rats were fed firstly in SPF Animal Department of Clinical Institute of China-Japan Friendship Hospital (occupancy permit: SYXK (Jing) 2005-0019). They were then randomized depending on body weight into 4 groups: the sham-operated group (normal control), the model group (blank control), the Zoledronic acid treated group (positive control) and the SGD treated group, 20 in each group (6 for radiographic studies and histological studies, 6 for RNA extraction and protein extraction, 8 for orthotopic studies).

### 2.4. Establishment of Bone Metastasis Model

The surgical procedure was the same as described previously [13]. Briefly, each animal was anesthetized with Pentobabital sodium (Merck, 45 mg/kg i.p.). A small incision was made over the anteromedial surface of the distal end of the tibia to expose the bone. A 22-gauge needle was used to bore a hole through the periosteum. Three microliters of MRMT-1 cells were injected into the tibia via a Hamilton syringe. The hole was closed using bone wax (Ethicon, USA), and the wound was closed with a metal clip. The procedure was identical for sham-operated animals except these injected with Hank's media alone. Animals were placed on a heated pad until they have regained consciousness and then returned to their home cages.

### 2.5. Drug Administration

As described previously [12], SGD was given to rats in the SGD group from day 1 to day 20 after modeling via gastric infusion, in dosage of 10 mL/kg per day (equivalent to 39 g of crude drug/kg per day), which was calculated by body surface area from the generally used dosage in clinical practice. 

Zoledronic acid (Novartis, Switzerland) was dissolved in water for injection to make 3 *μ*g/mL solution. It was given to rats in the Zoledronic acid positive control group at the 5th, 7th, 9th, 12th, 14th, 16th, and 19th day via intraperitoneal injection, 30 *μ*g/kg in each time [14].

As for rats in the sham-operated group and the model group, 10 mL/kg per day of deionized water was given orally. Each rat was monitored for changes in body weight and the dose of drugs was adjusted accordingly.

### 2.6. Radiological Analysis of Osteolytic Lesions

Hind limbs harvested at endpoint (day 21) were assessed radiologically prior to decalcification and histological staining (see later) using KXO-32R medical X-ray diagnosis system (TOSHIBA, Japan). Image Pro-Plus 7.0.1 software (Bethesda, USA) was utilized to quantify areas of osteolytic destruction [15].

### 2.7. Histological Evaluation of Tumor Volume and Osteoclast Activity

To identify osteoclasts, a tartrate-resistant acid phosphatase (TRAP) stain was used. Sections were stained for TRAP according to the manufacturer's instructions (Sigma, USA). Osteoclasts were counted either along the mineralised bone-tumor interface or along the mineralized bone-bone marrow interface. Tumor volume was evaluated as the percentage of tumor observed at 4x magnification in a field located contiguous to the growth plate [16].

### 2.8. Determining TRAP-5b Levels by ELISA Assay

The tibiae were sonicated in a PE buffer (CHAPS: GE Healthcare Life Sciences, China). The homogenate was centrifuged (10,000 g for 20 min at 4°C) and the supernatant were analyzed for protein content by the Bradford method. Serum and tissue levels of TRAP-5b were measured using ELISA kit (Immunodiagnostic Systems Ltd., Boldon, UK) according to the manufacturer's instructions. A standard curve was generated, and absolute concentrations were extrapolated from the standard curve. 

### 2.9. Analysis of TRAP mRNA by Quantitative Real-Time PCR

Total RNA was extracted from the tibiae using Trizol reagent (Invitrogen, USA), and first-strand cDNA was generated using the Superscript III reverse transcriptase system (Invitrogen, USA) according to the manufacturer's protocol. The real-time PCRs were performed in a final reaction volume of 25 *μ*L containing forward and reverse primers (0.15 *μ*M each), cDNA (10 × diluted), and 2 × SYBR Green PCR master mix (Toyobo, Japan). The real-time PCR specific primers were 5′-CAG CCC TTA TTA CCG TTT GC-3′ (forward) and 5′-GAA TTG CCA CAC AGC ATC AC-3′ (reverse) for TRAP, 5′-CTC AAC TAC ATG GTC TAC ATG TTC CA-3′ (forward) and 5′-CTT CCC ATT CTC AGC CTT GAC T-3′ (reverse) for GAPDH. Thermal cycles were 30 s at 95°C for initial denaturing followed by 40 cycles of 5 s at 95.0°C and 34 s at 60.0°C in ABI Step One sequence detection system (Applied Biosystems, USA) according to the manufacturer's instructions. The quantification data were analyzed with ABI Step One software, version 2.0 (Applied Biosystems, USA). The experiment was performed three times to achieve reproducibility. To confirm the amplification specificity, a melting curve analysis was added after thermocycling for every reaction, determining dissociation of the PCR amplified products from 60°C to 95°C. The mean value of the replicates for each sample was calculated and expressed as the threshold cycle (CT). The fold change in the level of TRAP, normalized by the level of GAPDH, was determined by the 2^−ΔΔCt^ method [17]. The 2% agarose gel electrophoresis from the other amplification designed with both of 28 cycles and final extension was 72°C for 10 min.

### 2.10. Orthotopic Implantation of MRMT-1 Cells

Cells were prepared as above and suspended in cold 50% Matrigel: PBS at a concentration of 2 × 10^7^ cells/mL. 100 *μ*L of the suspension was implanted subcutaneously at the mammary fat pad of 3 female SD rats (160 ± 10 g). Tumors harvested at day 14 were minced with scissors and 200 mg (approximately 4 × 10^6^ cells) were inoculated subcutaneously at the right dorsal flank of rats (*n* = 8) under pentobarbitone anesthesia [18]. Tumor size was measured in three dimensions by callipers to determine tumor volume and by tumor weight at euthanasia on day 28.

### 2.11. Statistical Analysis

All results are expressed as mean ± SEM (standard error of mean). Adopting SPSS 17.0 software, the intergroup comparison of measurement data was conducted by variance analysis. *P* < 0.05 was regarded as statistically significant.

## 3. Results

### 3.1. Radiological Analysis of Osteolytic Lesions

**Figure 2:**
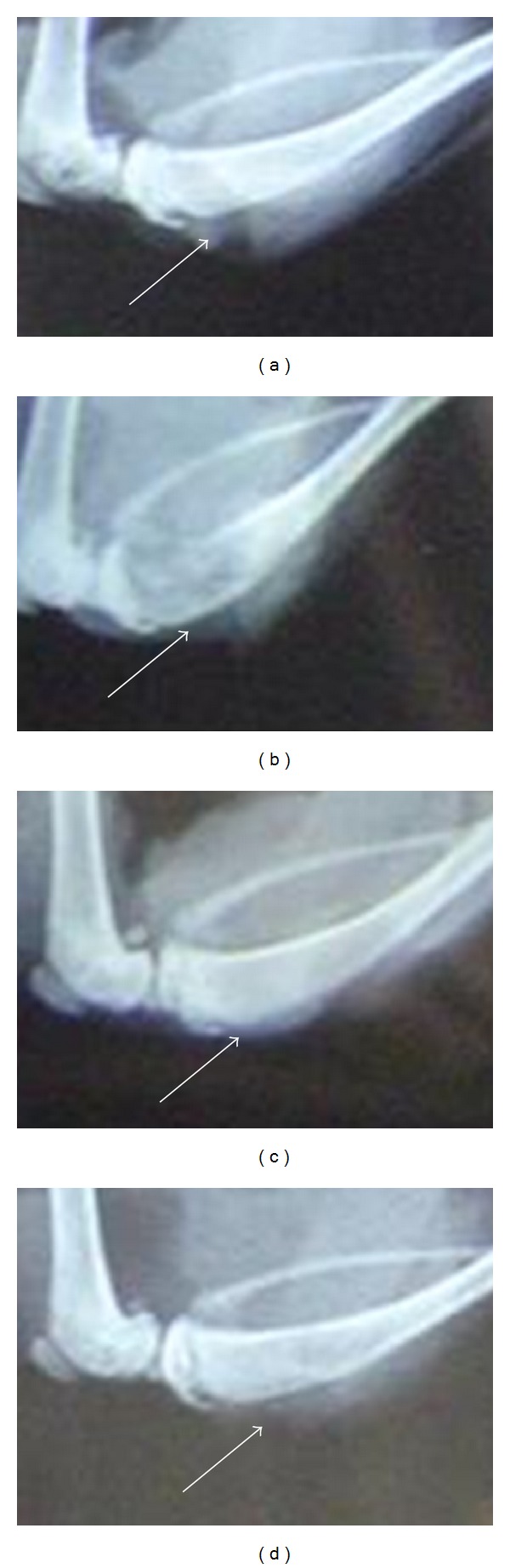
Radiographs of the structure of the bone and the development of the tumor. (a) Sham-op. group: no radiological change. (b) Tumor group: full thickness bicortical bone loss and displaced fractures were observed. (c) Zoledronic acid group and (d) SGD group: some loss of medullary bone and erosion of the cortical bone was apparent. Arrow indicates the site of injection.

As shown in Figure 2 and Table 2, normal structure of bone without any sign of destruction was shown in the sham-operated group. In contrast, significant destruction appeared in the other three groups (*P* < 0.01 versus normal control). The most severe damage revealed in the model group. As compared with the model group, osteolytic lesion areas were significantly smaller in Zoledronic acid or SGD treated bones (*P* < 0.01 versus blank control). Bone resorption was not found in the contralateral tibia of the 4 groups.

### 3.2. Histological Evaluation of Tumor Volume

As shown in Table 3, tumor volume was zero in the sham-operated group. In contrast, it comprised approximately 60% of the marrow cavity in tumor group (*P* < 0.01 versus normal control). Tumor volume did not exceed 30% of the marrow cavity in any animal in Zoledronic acid or SGD treated bones (*P* < 0.01 versus blank control).

### 3.3. Histological Evaluation of Osteoclast Activity

**Figure 3:**
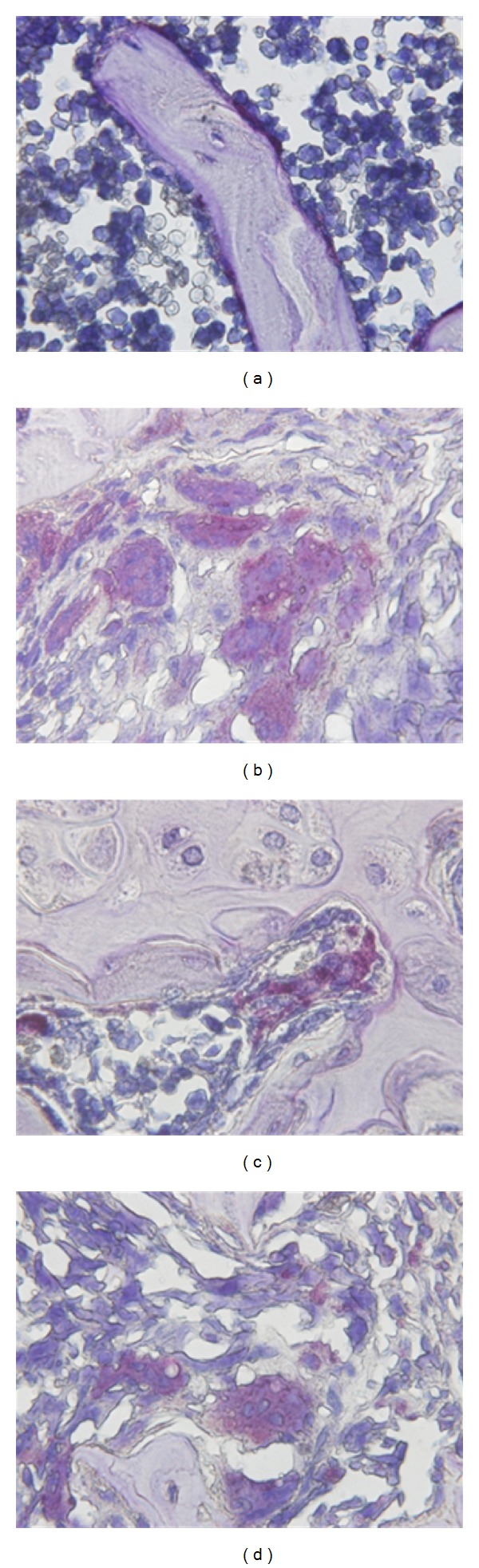
Effects of SGD on the proliferation of TRAP positive polykaryocytes (×1000). (a) Sham-op. group: TRAP positive polykaryocytes were rarely seen. (b) Model group: a large amount of scattered, strongly TRAP positive cells resided in the cancerous bone. (c) Zoledronic acid group and (d) SGD group: bone after treatment with Zoledronic acid or SGD contained only few, faintly stained TRAP positive profiles close to the bone.

TRAP is present in large polykaryocytes with multiple nuclei. TRAP positive profiles, which were associated with the bone and rare in normal control (sham-operated group) bone, appeared scattered in the tumor mass and close to the remnants of the trabeculae (Figure 3, Table 3). The overall number of TRAP positive polykaryocytes grew dramatically in model group (*P* < 0.01 versus normal control). In contrast, TRAP positive polykaryocytes were almost completely absent from sections obtained from Zoledronic acid or SGD treated bones (*P* < 0.05 versus blank control).

### 3.4. Serum Concentration of TRAP-5b

As shown in Table 4, serum concentration of TRAP-5b raised dramatically in the model group (*P* < 0.01 versus normal control). It decreased significantly after Zoledronic acid or SGD treatment (*P* < 0.01 versus blank control).

### 3.5. TRAP-5b Levels in Bone Tumor

As shown in Table 4, TRAP-5b levels increased significantly in the tibia of model group (*P* < 0.01 versus normal control). It decreased significantly after SGD treatment, (*P* < 0.05 versus blank control). Zoledronic acid treated bones showed a trend towards lower TRAP levels; however, the difference was not statistically significant.

### 3.6. TRAP mRNA

**Figure 4:**
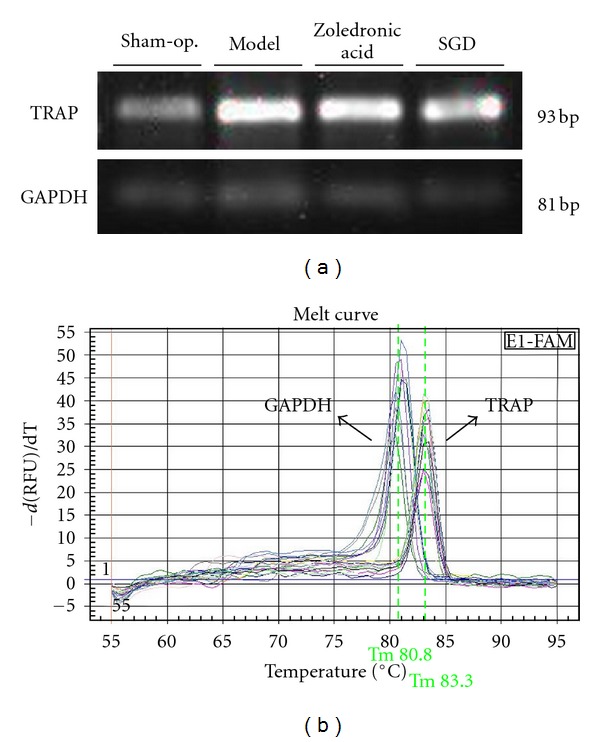
Expression of TRAP mRNA. (a) TRAP mRNA expression was assessed by RT-PCR, and GAPDH was used as control. (b) Melt curve analysis of TRAP and GAPDH. It demonstrates the single product-specific melting temperatures: TRAP (83.3°C), GAPDH (80.8°C). No primer-dimer formation was produced during 40 amplification cycles. The images were representative the littermates of the same litter from the four groups.

As shown in Table 4 and Figure 4, the mRNA expression of TRAP raised dramatically in the model group (*P* < 0.01 versus normal control). Zoledronic acid or SGD treatment significantly downregulated the expression of TRAP mRNA (*P* < 0.01 versus blank control).

### 3.7. Effects of SGD on Subcutaneous Tumors

Subcutaneous implantation of MRMT-1 breast cancer cells at the site of the mammary fat pad or the right dorsal flank resulted in successful establishment of palpable tumors. Tumor growth of MRMT-1 cells was fast in these locations, with a trend towards smaller tumors in SGD treated rats (Table 5). Endpoint wet weight of tumors in untreated rats showed a trend towards heavier tumors compared to SGD treated rats (Table 5); however, the difference was not statistically significant.

## 4. Discussions

TCM herbs have been used for several thousand years in China and partly demonstrated to have fewer side effects than chemical medicines to a certain extent. The current researches on the effects of TCM on osteoclasts' activity in BM are mostly based on clinical observations or in vitro experiment. In this study, the effect of TCM on osteoclasts activity was observed for the first time in a rat model of breast cancer bone metastasis.

According to TCM theories, Kidney (shen) main bone marrow of Health, the major pathogenesis of osteolytic bone cancer is “deficiency of Kidney Essence, reduction of marrow and flaccidity of bones”. Consequently, it is believed by TCM physicians that the “Nourishing Kidney to strengthen bone” action of TCM is beneficial to osteolytic bone destruction and useful for ameliorating bone cancer pain. SGD is a TCM prescription that has been applied in ameliorating cancer-induced bone pain for several decades. It is a typical “Nourishing Kidney to strengthen bone” decoction for the treatment of osteolytic bone destruction in bone cancer patients [12]. Owing to the complexity of the mixtures, the relationship and mechanism between safety and efficiency remain unclear.

SGD is consisted of three herbs and their chemical constituents are multiplex. In SGD, some chemical constituents, including psoralen from *Fructus Psoraleae* and assemble flavone of Drynaria rhizome from *Rhizoma Drynariae* were reported to stimulates osteoblasts proliferation and differentiation [19–23]. *Rhizoma Drynariae* dose dependently inhibited bone resorption in vitro [24]. Psoralen, isopsoralen, and quercetin, from *Fructus Psoraleae* and *Herba Speranskiae tuberculatae*, were reported to inhibit the proliferation of various carcinoma cell lines [25–28]. Scopoletin from *Herba Speranskiae tuberculatae* had antinociceptive effects in several pain models [29, 30].

In this rat model of breast cancer bone metastasis, bone destruction, changes in mineral content, and accumulation of TRAP positive polykaryocytes were all observed. TRAP positive cells were not only localized close to the bone but in addition they appeared uncharacteristically scattered within the body of the tumor. In both serum and bone tumors, TRAP-5b levels raised dramatically, which is produced exclusively by osteoclasts. In addition, the expression of TRAP mRNA in bone tumor increased significantly.

Semiquantification of X-ray showed that SGD treatment markedly reduced tumor-related osteolytic lesion of the bone. Furthermore, in histological studies the characteristic appearance of TRAP positive polykaryocytes observed in bone tumor was prevented by SGD treatment with a consequent reduction of osteoclastic activity. In addition, ELISA assay showed that both the serum concentration of TRAP-5b protein and TRAP-5b levels in bone tumor decreased significantly. Quantification of TRAP mRNA demonstrated that SGD also downregulated the expression of TRAP mRNA in bone tumor. The volume of bone tumor was decreased. Protective effects of SGD are localized to bone and do not affect subcutaneous tumors.

However, the effective constituents of SGD and the mechanisms of action are worth investigating in the future. Tumor-induced osteolysis is due to recruitment and activation of osteoclasts resulting from interactions between receptor activator for nuclear factor-*κ*B (RANK) on osteoclasts and precursors and the RANK ligand (RANKL) on osteoblasts and stromal cells [31–33]. More studies are needed to further confirm the effectiveness of SGD on osteoclasts inhibiting factor and activating factor, such as osteoprotegerin (OPG) and receptor activator for nuclear factor-*κ*B ligand (RANKL). 

## 5. Conclusions

In summary, our data are the first to show in well-controlled bone metastasis animal models that SGD has significant antiosteoclasts effect, that could be directly related to its TRAP inhibit activity. In addition it prevented bone tumor proliferation in bone cancer models.

## Figures and Tables

**Table 1:** Constituents of Sangu Decoction.

Chinese name	Botanical name	Weight (g)	Voucher numbers
Buguzhi	*Fructus Psoraleae *	15	NCPS00601
Gusuibu	*Rhizoma Drynariae *	15	NCPS00602
Tougucao	*Herba Speranskiae tuberculatae *	9	NCPS00603

**Table 2:** Comparison of osteolytic lesion areas in different groups (*n* = 6).

Group	Osteolytic lesion area (mm^2^)	
	Ipsilateral tibia	Contralateral tibia
Sham-op.	0.00 ± 0.00**	0.00 ± 0.00
Model	11.90 ± 0.56^∆∆##^	0.00 ± 0.00
Zoledronic acid	3.37 ± 0.70^∆∆∗∗##^	0.00 ± 0.00
SGD	7.01 ± 0.33^∆∆∗∗##^	0.00 ± 0.00

Notes: ^∆^ *P *< 0.05, ^∆∆^ *P *< 0.01, compared with the sham-operated group; *P < 0.05, **P < 0.01, compared with the model group; ^#^ *P *< 0.05, ^##^ *P *< 0.01, compared with the contralateral tibia.

**Table 3:** Comparison of osteoclasts and tumor volume in different groups (*n* = 6).

Group	TRAP (+) cells/HP	Tumor volume (%)
Sham-op.	1.88 ± 2.92**	0.00 ± 0.00**
Model	40.84 ± 25.59^∆∆^	61.67 ± 12.11^∆∆^
Zoledronic acid	14.18 ± 13.14^∆∆∗^	27.50 ± 3.62^∆∆∗∗^
SGD	15.75 ± 3.10^∆∗^	29.00 ± 6.03^∆∆∗∗^

Notes: ^∆^ *P *< 0.05, ^∆∆^ *P *< 0.01, compared with the sham-operated group; *P < 0.05, **P < 0.01, compared with the model group.

**Table 4:** Comparison of TRAP-5b protein levels and TRAP mRNA in different groups (*n* = 6).

Group	TRAP-5b (U/L)		TRAP mRNA (2^−ΔΔCT^)
	Serum	Ipsilateral tibia	Ipsilateral tibia
Sham-op.	0.83 ± 0.21**	0.13 ± 0.01**	0.98 ± 0.07**
Model	1.22 ± 0.21^∆∆^	0.55 ± 0.06^∆∆^	35.43 ± 0.86^∆∆^
Zoledronic acid	0.74 ± 0.23**	0.50 ± 0.15^∆^	20.11 ± 0.75^∆∆∗∗^
SGD	0.85 ± 0.17**	0.28 ± 0.10*	21.34 ± 1.60^∆∆∗∗^

Notes: ^∆^ *P *< 0.05, ^∆∆^ *P *< 0.01, compared with the sham-operated group; *P < 0.05, **P < 0.01, compared with the model group.

**Table 5:** Comparison of tumor volume and tumor weight in different groups (*n* = 8).

Group	Tumor volume (cm^3^)	Tumor weight(g)
Sham-op.	0.00 ± 0.00	0.00 ± 0.00
Model	24.11 ± 19.84^∆∆^	21.71 ± 7.71^∆∆^
Zoledronic acid	16.56 ± 14.95^∆∆^	23.14 ± 17.64^∆∆^
SGD	15.41 ± 8.95^∆∆^	13.00 ± 8.54^∆∆^

Notes: ^∆^ *P *< 0.05, ^∆∆^ *P *< 0.01, compared with the sham-operated group; *P < 0.05, **P < 0.01, compared with the model group.
